# Change-Point Detection of Peak Tibial Acceleration in Overground Running Retraining

**DOI:** 10.3390/s20061720

**Published:** 2020-03-19

**Authors:** Pieter Van den Berghe, Maxim Gosseries, Joeri Gerlo, Matthieu Lenoir, Marc Leman, Dirk De Clercq

**Affiliations:** 1 Biomechanics and Motor Control of Human Movement, Department of Movement and Sports Sciences, Ghent University, 9000 Ghent, Belgium; Maxim.Gosseries@ugent.be (M.G.); Joeri.Gerlo@ugent.be (J.G.); Matthieu.Lenoir@UGent.be (M.L.); Dirk.DeClercq@ugent.be (D.D.C.); 2 IPEM, Department of Arts, Music and Theatre Sciences, Ghent University, 9000 Ghent, Belgium; Marc.Leman@ugent.be

**Keywords:** biomechanics, augmented feedback, motor learning, gait adaptation, music

## Abstract

A method is presented for detecting changes in the axial peak tibial acceleration while adapting to self-discovered lower-impact running. Ten runners with high peak tibial acceleration were equipped with a wearable auditory biofeedback system. They ran on an athletic track without and with real-time auditory biofeedback at the instructed speed of 3.2 m·s^−1^. Because inter-subject variation may underline the importance of individualized retraining, a change-point analysis was used for each subject. The tuned change-point application detected major and subtle changes in the time series. No changes were found in the no-biofeedback condition. In the biofeedback condition, a first change in the axial peak tibial acceleration occurred on average after 309 running gait cycles (3′40″). The major change was a mean reduction of 2.45 g which occurred after 699 running gait cycles (8′04″) in this group. The time needed to achieve the major reduction varied considerably between subjects. Because of the individualized approach to gait retraining and its relatively quick response due to a strong sensorimotor coupling, we want to highlight the potential of a stand-alone biofeedback system that provides real-time, continuous, and auditory feedback in response to the axial peak tibial acceleration for lower-impact running.

## 1. Introduction

The peak tibial acceleration of the axial component can be defined as the maximum positive value of the signal during stance. The axial peak tibial acceleration is considered a surrogate measure for impact loading and can be registered by an accelerometer [1,2,3]. Peak tibial acceleration has been used as input to biofeedback systems [4,5]. These biofeedback systems can provide acoustic signals scaled to the magnitude registered by a shin-mounted accelerometer. The peak tibial acceleration could be lowered during running on a treadmill with real-time auditory and/or visual biofeedback compared with running without the biofeedback [4,6,7,8]. Lowering the axial peak tibial acceleration in runners experiencing high-impact loading has been done with the goal of reducing the risk of running-related injuries [9,10,11]. These findings highlight the potential of an individualized approach of gait retraining using augmented feedback on peak tibial acceleration in real time. A drawback of the studies on running retraining using biofeedback, next to the treadmill setup, is that a limited amount of steps were analyzed for each recording period (e.g., 20 in [4]) (Table 1). As a result, our knowledge of the time course of changes in the targeted biomechanical signal is not yet understood. Therefore, a wearable biofeedback system that continuously collects tibial acceleration was recently developed and utilized [5,12]. This system was developed to continuously record tibial acceleration at a high sampling rate and to immediately detect the magnitude and the time of the peaks of the axial component [12]. Under supervised use, a reduction of almost 30% in axial peak tibial acceleration was found when comparing the end of a 20-min biofeedback run with the no-biofeedback condition [5]. Given that the inter-subject response to a reduction in axial peak tibial acceleration can vary [7], one might expect an individual evolution in magnitude and presumably also in the timing of the change next to the evolution of the whole group. Although the technical aspect of augmented feedback systems is developing rapidly (Table 1), little attention has been paid to when or how people interact with biofeedback on a running gait parameter [13,14], while this is imperative for understanding motor adaptations induced by the feedback parameter. For example, a first session of gait retraining may comprise a half-hour of running, whereas a major change in the desired performance may already be achieved after several minutes. Therefore, timing values are valuable for the design of gait retraining programs.

The transition to a running technique involving less axial peak tibial acceleration is a process of motor learning, which may occur in stages [15]. Inspection of separable stages allows the design of experiments with higher specificity for certain aspects of that process [15]. Desired elements of a movement can be learned at different rates [15,16], meaning that motor skill improvement can vary between subjects. As such, the profile (location and duration) of evolution in axial peak tibial acceleration may also vary between runners initiating gait retraining. The gait retraining studies providing unimodal (i.e., auditory or visual) biofeedback on the axial peak tibial acceleration have focused on the early adaptation phase of running with less peak tibial acceleration. The change(s) and the variability in peak tibial acceleration inherent to this locomotor task have been neglected within a session [4,5,6,8]. A reason to neglect the time course of the axial peak tibial acceleration may be that relevant changes in such a signal are usually not easily discernible by sight. The technique of change-point analysis may be of use to detect event(s) at which the underlying dynamics of a signal changes over time [17,18,19,20,21,22,23]. Several types of control statistics have been used for change-point discovery. For example, control charting provides upper and lower bounds of an individual chart with the assumption that no change has occurred [24]. Change-point analysis may be more powerful to detect relatively small or sustained shifts from the average because it better characterizes the time at which a change began to occur, controls the overall error rate, and is easily applicable for time series segmentation [25].

We employed a change-point application with tuned parameters to evaluate the time course of the axial peak tibial acceleration in the early adaptation phase of biofeedback-driven gait retraining. As subjects were expected to respond differently to the biofeedback-driven approach of gait retraining, a typical analysis of group data might have masked individual changes. Therefore, a single-subject analysis was employed to identify when runners shifted their axial peak tibial acceleration in the early adaptation phase of gait retraining.

## 2. Materials and Methods

### 2.1. Subjects

Following an initial screening session, ten runners (five males and five females, body height: 1.70 ± 0.07 m, body mass: 67.7 ± 7.4 kg, age: 33 ± 9 years) with high axial peak tibial acceleration impacting the lower leg (at least 8 g at 3.2 m·s^−1^, mean ± SD: 11.1 ± 1.8 g) participated in our study. This sample size is in line with previous studies using biofeedback to stimulate running with less impact loading (i.e., lower-impact running) [4,8]. Requirements for participation were to run ≥ 15 km/week in non-minimalist footwear and to be injury free for ≥ 6 months preceding the experiment [26]. Thus, the subjects reported running 29 ± 12 km per week at 2.88 ± 0.31 m∙s^−1^ (mean ± SD). The cohort consisted of nine rearfoot strikers and one forefoot striker (Appendix A), categorized using plantar pressure measurements characterized by high temporal and spatial resolution [27]. All subjects signed an informed consent approved by the ethical committee upon participation (Bimetra number 2015/0864).

### 2.2. Intervention

**Figure 1:**
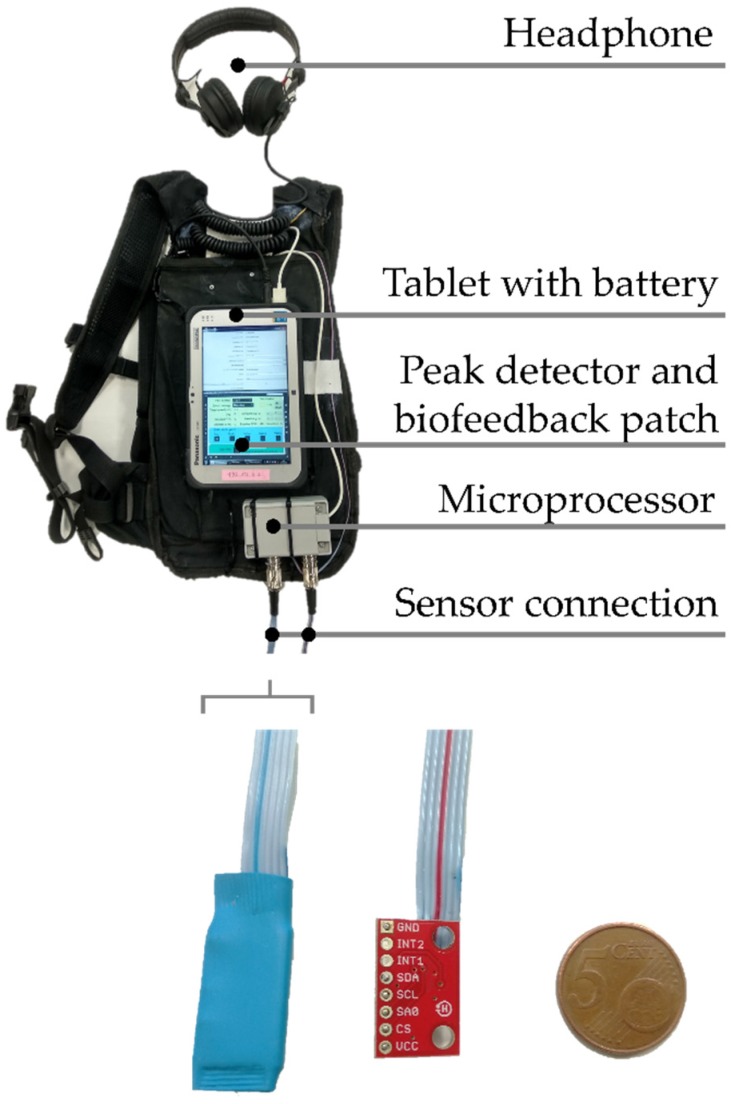
(upper panel) Picture from the instrumented backpack. (lower panel) The accelerometer (left) with and (middle) without the shrink sleeve and (right) a 5-cent piece.

The subject was equipped with a stand-alone backpack system, connected to two lightweight sensors. The backpack system was developed for real-time auditory feedback with respect to peak tibial acceleration in overground running environments. The main components are indicated in Figure 1. The sensor was powered using a battery that allowed long uninterrupted runs, in order to use the least amount of power possible. The sensor of interest was a low-power MEMS three-axis accelerometer (Sparkfun, Boulder, CO, USA). The accelerometer characteristics were as follows: mass: 20 milligrams, resolution: 70 mg, with digital output (SPI-compatible). The breakout board (dimensions: 21 × 13 mm) was fitted in a shrink socket [12]. The total mass was less than 3 grams, making it lighter than commercially available sensors in a plastic housing that have been used for the registration of tibial acceleration during running [2,3,4]. A very lightweight accelerometer is beneficial because it is less susceptible to unwanted secondary oscillations due to inertia. The accelerometer of interest had dynamically user-selectable full scales of ±6 g/±12 g/±24 g and was capable of measuring accelerations with output data rates from 0.5 Hz to 1 kHz [28]. Provot and colleagues [2] recommended a sampling rate of at least 400 Hz for tests involving the measurement of tibial acceleration during running activities. A sampling rate of 1000 Hz was selected because lower rates might have caused the actual value of the peak to be missed. The tibial acceleration was continuously measured. The axial component was chosen for analysis because tibial acceleration has typically been analyzed unidirectionally [3,6,7,8,9,29,30] and because it has been associated with a history of tibial stress fracture in distance runners [30]. If the signal range exceeds the capture range of the sensor, the measured signal is clipped at the extremities [1]. The highest value of axial peak tibial acceleration registered in a previous study with the system while running on a sports floor was 12.4 g at the same running speed compared to the present experiment. Therefore, we expected the accelerometer to have a sufficient range (±24 g) to prevent clipping while running overground at 3.2 m∙s^−1^. Post hoc inspection of the values of axial peak tibial acceleration revealed that the selected measurement range was more than enough for the goal of our study.

**Figure 2:**
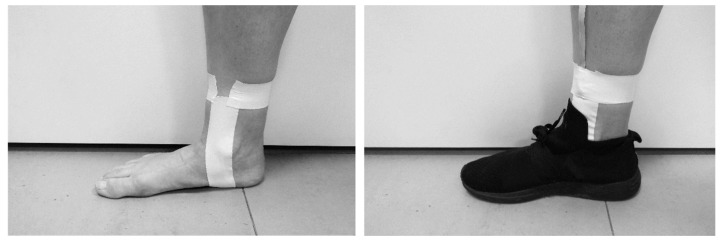
Attachment method of the sensor to the lower leg. (left) Pretension is applied to the skin near the site of attachment. (right) The accelerometer is firmly fixed with tape.

The tibial skin was prestretched bilaterally at ~8 cm superior to each medial malleolus to minimize skin oscillation [9,12]. An illustration of such prestretch through the use of zinc oxide tape (Strappal, Smith and Nephew, UK) is shown in Figure 2. Each accelerometer was placed on the tight skin of the prestretched area. The axial axis of an accelerometer was visually aligned with the longitudinal axis of each shin while the subject was standing [12,29]. The distal aspect of both lower legs was locally wrapped in a non-elastic adhesive bandage (Strappal) [12]. The manner of attachment with visual alignment and taping of the sensor to the skin has been applied in research on tibial acceleration in running [9,12]. The simple mounting technique has resulted in repeatable mean values of the tibial shock between running sessions [12], even without highly accurate standardization. The total mass of the stripped backpack with the electronic components strapped to the inside shell was equal to 1.6 kg. The same backpack has been used in previous studies intertwining locomotion and music (e.g., [13,29,30]). Subjects wore their habitual running footwear to reflect the usual running habits and to increase the ecological validity of the study. A passive noise-canceling headphone was worn.

**Figure 3:**
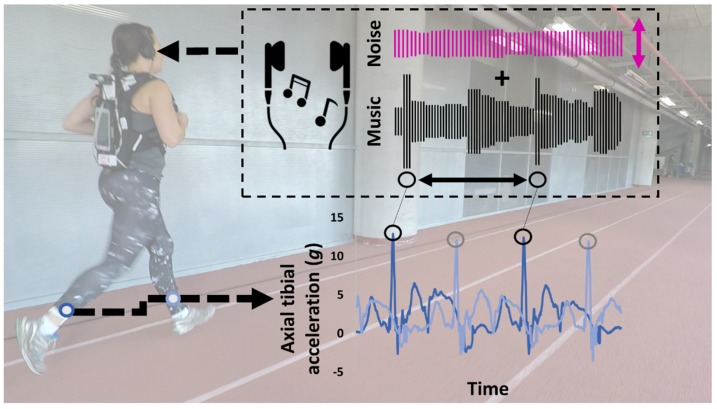
A subject running indoors on an athletic track (287 m/lap) at the instructed speed of ~3.2 m·s^−1^. Real-time auditory biofeedback in response to the axial peak tibial acceleration was provided by a wearable interactive system to the runner with high axial peak tibial acceleration. The sensor processing involved real-time peak detection. The music processing comprised tempo synchronization of the music combined with peak-based noise added to the music playing.

The running session was performed on an athletic track at an indoor training facility (Figure 3) (Video S1, Supplementary Materials). The session consisted of a no-biofeedback condition and a biofeedback condition, representing the control and experimental conditions, respectively. Accelerometer data were acquired with real-time detection of the magnitude and the timing of axial peak tibial acceleration [5]. The no-biofeedback condition was a warm-up run of 4.5 min at the instructed speed of 3.2 ± 0.2 m·s^−1^. In the case of bilateral elevation of axial peak tibial acceleration, the leg with the highest value was addressed in the retraining [8]. Thereafter, auditory biofeedback on axial peak tibial acceleration was continuously provided in real time. Biofeedback helps to develop the connection between the extrinsic feedback and the internal sensory cues associated with the desired motor performance during the first phase of motor retraining (i.e., the early adaptation phase) [10]. A patch was designed in Max MSP software (v7, Cycling’74, San Francisco, CA, USA) to provide the auditory biofeedback [13]. The concurrent auditory feedback consisted of a music track that was continuously synchronized to the step frequency of a runner. A music database consisting of 77 tracks with a clear beat in a tempo range of endurance running was preselected. D-Jogger technology was employed to continuously align the beats per minute of the music to the steps per minute of the runner [31]. When step frequency changed by > 4% in steps per minute for 8 s, another song from which the beats per minute better matched the steps per minute automatically started playing. The biofeedback consisted of pink noise that was superimposed onto the music. Importantly, the noise’s intensity was perceivable and depended on the magnitude of axial peak tibial acceleration [5,13]. The past five values of axial peak tibial acceleration were averaged through a 5-point moving average [9]. Thus, the wearable system detected the peak tibial acceleration and compared the selected gait parameter over a window of several strides with respect to a relative threshold value. The noise was added whenever that value exceeded a predetermined threshold of approximately 50% of the baseline value in the no-biofeedback condition. The chosen target was similar to previous gait retraining studies [6,7,8,9]. Six levels of noise loudness were empirically created for good discretization [13]: % noise, % baseline axial peak tibial acceleration: 100%, >113%; 80%, 96%–113%; 60%, 80%–95%; 40%, 65%–79%; 20%, 48%–64; 0%, <48%. The noise loudness was calculated as a percentage of the root mean square of the amplitude level of the music. Only synchronized music was provided when the momentary axial peak tibial acceleration of the runner was below the threshold target. The baseline value of axial peak tibial acceleration was the mean axial peak tibial acceleration of 90 s in the no-biofeedback condition.

A self-discovery strategy was elicited. Each runner was instructed to find a way to run with less axial peak tibial acceleration by increasing the musical quality (i.e., lowering the noise loudness level), although no instructions were given on how to achieve this [4,9]. Subjects subsequently ran for 20 min in total, separated by a short technical break after 10 min to check the software. The instructions were repeated during the break. The running speed was monitored by a chronometer to provide verbal feedback on a lap-by-lap basis. Acceleration data of one subject were not recorded during the second half of his warm-up.

### 2.3. Data Processing

All detected axial peak tibial accelerations (*n* = 18,529) were preprocessed using custom MATLAB scripts [12]. The data of the no-biofeedback condition (1.5 min, baseline) were concatenated with the data of the biofeedback condition (2 time periods of 10 min for the change-point analysis). The first 90 s were composed of the no-biofeedback condition. The values have been deposited in a public repository [32]. Because all subsequent values were part of the time series, we could determine the timing and the duration of a change. Change-Point Analyzer (v2.3, Taylor Enterprises, Libertyville, IL, USA) was employed for each subject to detect individual changes in the axial peak tibial acceleration over time. The analysis tool has been previously used in health sciences to determine if and when statistically significant changes in 1D time series occurred [33,34]. The procedure proposed by Taylor [24,35] for performing the change-point analysis uses a combination of cumulative sum charts and serial bootstrap sampling. Both the application of cumulative sum charts [33,34] and the application of bootstrapping [35] have been suggested for the problem of detecting a single change [25]. The procedure combines these two approaches, whereby Change-Point Analyzer allows multiple changes to be detected iteratively in a time series. In essence, this technique searches across the time frames looking for changes in the values that are so large that they cannot reasonably be explained by chance alone [36]. We refer to Appendix B for a more detailed description of the change-point analysis that is based on a statistical mean-shift model. The changes are accompanied by associated confidence levels and confidence intervals for the times of the changes. The following configuration was applied in the Change-Point Analyzer: the confidence level for the time interval of changes: 95%; the number of bootstraps: 1000; randomization without replacement; mean square error-based time estimates; groups of 33 rows. As such, no assumptions were violated. Importantly, the confidence level for candidate changes and for the inclusion of changes was set at 95% and 99%, respectively. The no-biofeedback functioned as the control condition, so we assumed this time period to be steady-state. The default confidence levels were set at 50% and 90%, which would have led to a false identification of change points in the time period of no-biofeedback. Thus, these levels were upscaled to increase the likelihood of detecting valid change points that represented a valuable mean shift in the time series of interest. We examined the individual changes in the axial peak tibial acceleration, being an increase or a decrease, accompanied by its confidence interval and location, the lowest zone of axial peak tibial acceleration and its duration, and the (change in) standard deviation of the grouped signal. The signal variability reported throughout this paper is always a long-term variability on the grouped signal of 33 consecutive axial peak tibial accelerations. The estimated standard deviation of the grouped signal is based on the whole running session by concatenating both running conditions and by considering the change in the signal. The timing of occurrence and the magnitude of the detected first and major change points were averaged for our cohort of runners with high axial peak tibial acceleration. The occurrence of change is expressed in terms of running gait cycles (strides) or in units of time.

## 3. Results

All subjects discovered a way to run with less axial peak tibial acceleration. No change point was detected in the no-biofeedback condition. At least one change point was detected for each subject in the biofeedback condition (Table 2), meaning that the runners swiftly reacted to the real-time auditory biofeedback. The first change of -1.26 ± 2.59 g in axial peak tibial acceleration was found after 309 ± 212 running gait cycles (3′40″ ± 2′24″) of running with biofeedback. This first change did not correspond to the major change in eight out of ten runners. The major change in axial peak tibial acceleration was consistently a reduction in axial peak tibial acceleration of 2.45 ± 1.99 g. The major change was found after 699 ± 388 running gait cycles (8′04″ ± 4′38″). 

**Figure 4:**
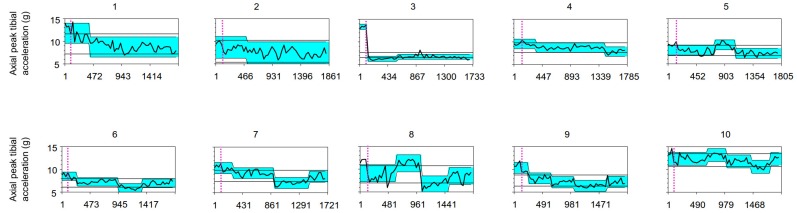
Graphical representation of the results of the change-point analysis. The graph in each panel depicts the temporal evolution in axial peak tibial acceleration of a subject (numbered 1 to 10) for the concatenated conditions of no-biofeedback (baseline) and biofeedback. A detected change point in the response to biofeedback is represented by a slanting line with a shift in the shaded background. When one or more change points were detected, the time series of the runner’s axial peak tibial acceleration became divided into smaller segments. The control limits (horizontal lines) assume the values come from the normal distribution. The first 90-s or 127 ± 10 (mean ± SD) running gait cycles belong to the no-biofeedback condition. X-axis: running gait cycles (strides). Y-axis: the axial peak tibial acceleration value in g.

As expected, the location of detected change points varied considerably between runners (Figure 4). For example, in subject 1 the real-time biofeedback resulted in a fast, substantial, and sustained reduction in axial peak tibial acceleration throughout the intervention. Following an initial reduction, eight subjects further shifted (further decline or slight increase) in axial peak tibial acceleration. After reaching a temporary minimum in axial peak tibial acceleration, its magnitude slightly increased for six subjects but remained below the baseline. The first change in axial peak tibial acceleration, which also induced the zone of lowest axial peak tibial acceleration, was sustained by two subjects until the end of the biofeedback condition. Most subjects further adapted in the biofeedback condition. No significant change was detected for the standard deviation of the averaged values over 33 grouped axial peak tibial accelerations, indicating no discernible long-term variability in axial peak tibial acceleration of a runner during the early adaptation process of lower-impact running.

## 4. Discussion

We present a simple method to detect changes in the time course of a biomechanical signal when runners engage in overground gait retraining. As such, we could provide strong empirical evidence when runners changed their axial peak tibial acceleration in response to real-time auditory biofeedback on it. An interactive feedback device was used that modulated the runner’s system dynamics in a self-discovery manner without giving specific instruction on running gait (i.e., “land softer” [6,7,8]). For that aim, we used a reinforcement learning paradigm for biofeedback control in which less axial peak tibial acceleration maximizes the positive reward (i.e., clear sound and synchronized music) and minimizes the negative reward (i.e., noise added to synchronized music). Without explicit cued instructions for an altered running technique, the chosen auditory biofeedback can influence the ongoing running style due to strong auditory–motor couplings in the human brain, thereby providing an avenue for a shift in musculoskeletal loading that may be beneficial to reduce running-related injuries. 

**Figure 5:**
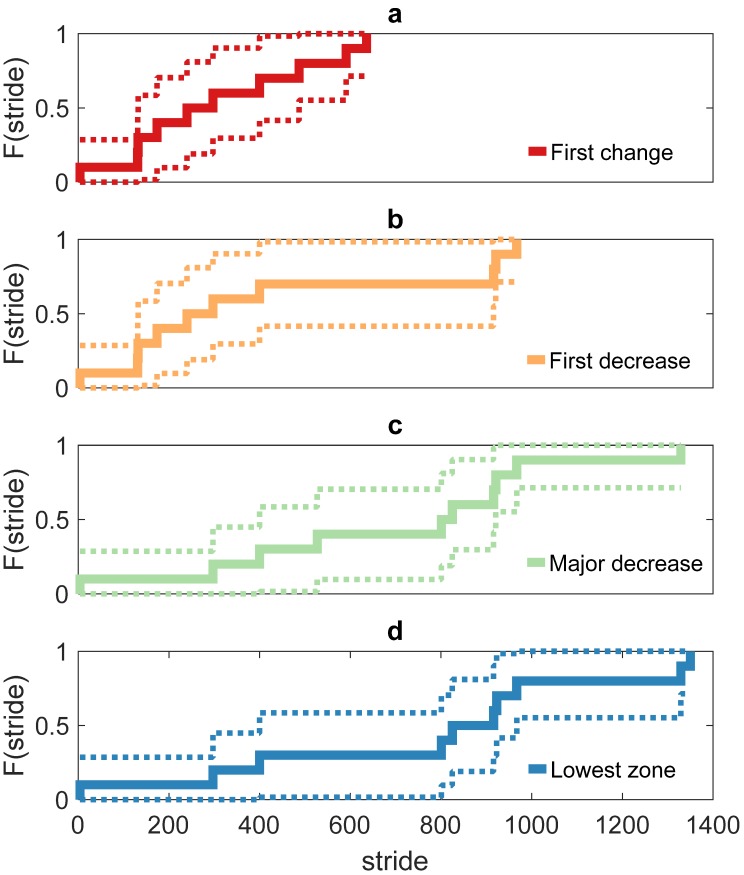
The cumulative distribution function describing (**a**) the first change in axial peak tibial acceleration, (**b**) the first reduction, (**c**) the major change, and (**d**) the zone of the lowest axial peak tibial acceleration in the biofeedback condition. In each panel, the horizontal axis shows the number of the gait cycles (strides) and the vertical axis shows the cumulative probability (F(stride)) between zero and one. The dashed lines indicate the Greenwood confidence interval.

In the early adaptation phase of lower-impact running, runners with high axial peak tibial acceleration reacted differently in time and in magnitude to the auditory biofeedback that stimulated lower-impact running. The inter-subject variation in time to the changes in axial peak tibial acceleration during the intervention highlights the relevance of the single-subject analysis. A first swift change demonstrates the ability of humans to react relatively fast to an auditory biofeedback stimulus on a modifiable outcome parameter of running gait. The major reduction in axial peak tibial acceleration was generally found after about 8 min of biofeedback with no change in grouped signal variability. In general, such a short time frame might suffice to successfully explore a biofeedback-driven style of lower-impact running. Considerable variation in the time to the major reduction in axial peak tibial acceleration (4 to 1329 gait cycles) was, however, noticed among the high-impact runners. Our data seem to suggest a possible distinction between slow and fast gait adapters based on biomechanical, physiological, and motor control determinants. The inter-subject variance in the profile of change may be due to the individualized motor retraining approach, through auditory biofeedback on an outcome parameter, whereby numerous (combinations of) gait adaptations might result in a reduction of the axial peak tibial acceleration. The inter-subject variation in this group of high-impact runners is further illustrated in Figure 5. The empirical cumulative distribution function was created using the Kaplan–Meier estimator to approximate the distribution of the time to the detected changes. 

The group was able to temporarily reduce the axial peak tibial acceleration to a minimum zone of 68% compared with running without biofeedback. It is debatable whether an extreme target of −50% in axial peak tibial acceleration [6,7,8,9], which was generally too hard to achieve or to maintain, is required in the early adaptation phase of biofeedback-driven running retraining. Furthermore, not all high-impact runners could maintain their major reduction throughout the session. Full retainment of the major reduction in axial peak tibial acceleration may depend on the mental and physical loads required to handle the auditory–motor coupling at the instructed running speed, the target of reduction in peak tibial acceleration, and/or the specific task dealing with implicit motor learning. A more realistic target for the targeted population seems to be -30% in axial peak tibial acceleration, which will also reinforce the reward of running with music only (i.e., no noise). This is in agreement with the recent finding of runners experiencing high axial peak tibial acceleration who were able to achieve and maintain a reduction of about 30% of its magnitude after completing a retraining program in the laboratory [11]. 

Individual long-term variability in axial peak tibial acceleration did not change when a state of lower-impact running was achieved by the applied configuration and measurement techniques. The impending change(s) in the movement pattern induced a similar variability in the axial peak tibial acceleration. Hence, variability in the magnitude of the axial peak tibial acceleration is inherent to both high- and lower-impact running when engaging in biofeedback-driven gait retraining. If we assume the axial peak tibial acceleration to be an expression of motor coordination, then its consistent variation at the end of the biofeedback condition suggests a stable running pattern, even a new phase in the motor learning process. 

Due to a lack of a retention test following the intervention, the adaptation phenomenon cannot be linked to a learning effect. We do not yet know whether this retraining results in a stable and lasting reduction in the axial peak tibial acceleration in the long term. Nonetheless, early adaptation is the first step towards feasible motor retraining outside the laboratory. Caution is required when interpreting our results. The biofeedback run was paused after 10 min while the verbal instructions were repeated. This intervention may have influenced the observed learning rate in the retraining session. Overall, we believe that, based on our findings, a change-point analysis can be employed to determine when runners start responding to real-time biofeedback that stimulates lower-impact running. Next to the simple method of change point detection in a biomechanical signal (i.e., axial peak tibial acceleration), our experimental work aids in understanding the human dynamic system and its adaptive control of movement over time. The understanding of the adaptation to running overground with a wearable auditory biofeedback system is one of the many steps in the evolution toward evidence-informed use of wearable technology in daily life. Similar to Moens and Lorenzoni and colleagues [13,31], none of the subjects complained about the stripped backpack. Nevertheless, the weight of the system could be trimmed and a higher level of comfort could be simultaneously achieved by opting for a backpack commonly used in trail running, which may be filled with a slim processing unit that permits wireless data transfer. Furthermore, smart textiles could enhance the standardization of the sensor’s location and orientation. While the applied system has been proven reliable both within sessions and between them using simple mounting principles [12], embedding a wireless accelerometer in a leg compression sleeve may further improve the reliability of the measurement of axial peak tibial acceleration between sessions. An improvement on the biofeedback side may be to replace an arbitrary level of change by a detected change. The offline analysis following data collection may be a stepping stone to the development of an online detection of change points. An online detection during the gait retraining has not yet been explored, but may permit to steer the noise loudness levels according to the abilities of a subject instead of being bound to preconfigured levels. Future research could develop online change point detection to better steer and individualize the level of biofeedback.

In this paper, we provide an extension of previous works related to gait retraining using real-time biofeedback with respect to peak tibial acceleration. The main contributions of this paper compared to previous works are the evaluation of results in a different running environment and the implementation of change point detection for a particular biomechanical signal. On the one hand, this study provides a motivational approach, through the use of synchronized music, to transition biofeedback-driven running retraining from the laboratory to the field. Efforts were made to enable continuous sensing of and feedback on peak tibial acceleration in order to go beyond the traditional laboratory setting. The wearable system drives lower-impact running by reducing the peak tibial acceleration of overground running versus running without a device. On the other hand, the simple-to-use application enables a subject-specific evaluation of adaptive changes in peak tibial acceleration during the biofeedback-driven gait retraining in time. Because of the swift reduction in axial peak tibial acceleration when initiating gait retraining, we want to highlight the potential of a stand-alone biofeedback system and its strong sensorimotor coupling.

## Figures and Tables

**Table 1:** Milestones illustrating the technological advancements and feedback modalities on augmented feedback with respect to tibial acceleration. The eye and ear icons indicate visual and auditory feedback, respectively.

Studies	Hardware for Biofeedback	Feedback Modality	Running Environment	Trials for Analysis
Crowell et al. (2010)	1 × accelerometer 1 × computer 1 × monitor screen	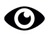	Treadmill, laboratory	20 averaged per condition
Clansey et al. (2014)	1 × accelerometer 1 × computer 1 × projection screen 1 × speaker set	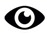 + 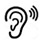	Treadmill, laboratory	6 averaged per condition
Wood and Kipp (2014)	1 × accelerometer 1 × computer with speakers	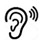	Treadmill, laboratory	20 averaged per condition
Present study	2 × accelerometers 1 × instrumented backpack 1 × headphone	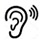	Overground, athletic facility	1853 ± 88 (mean ± SD) in total

**Table 2:** Detected change points in the runners with high axial peak tibial acceleration (APTA). Each row represents a subject. Subjects are sorted according to the number of detected change points, and then, according to the timing of the first change in APTA. The individual location corresponds to the detected APTA in the biofeedback condition. The + and – signs indicate an increase and a decrease, respectively, in the APTA. ^a^ indicates the change in the APTA signal that corresponds to the major decrease in magnitude as identified by the Change-Point Analyzer. The estimated standard deviation of the grouped APTA is based on the whole running session.

ID	APTA (g) Baseline	Number of Change Points	Location of the Change Point	95% Confidence Interval	Δ Change Inter-Segments in APTA (g)	Zone of Lowest APTA (g) {% vs. Baseline}	Estimated Standard Deviation
1	13.21	1	297 ^a^	231–330	−3.04	8.75 {66%}	0.75
2	9.66	1	400 ^a^	235–631	−1.24	7.44 {77%}	0.81
3	13.43	2	4 ^a^ 466	4–4 367–1555	−7.05 +0.42	6.30 {47%}	0.19
4	9.40	2	240 1329 ^a^	240–306 1263–1362	−0.81 −0.90	7.86 {84%}	0.35
5	9.28	2	636 967 ^a^	373–703 934–967	+1.17 −1.90	7.33 {79%}	0.37
6	8.87	3	132 825 ^a^ 1221	66–165 825–858 1188–1254	−1.24 −1.28 +0.99	5.96 {67%}	0.31
7	10.83	3	174 801 ^a^ 1329	75–273 768–801 1296–1362	−1.12 −2.14 +1.36	7.14 {66%}	0.40
8	11.83	3	487 916 ^a^ 1378	190–520 916–916 1345–1477	+2.18 −4.64 +1.86	6.65 {56%}	0.63
9	11.03	4	131 527 ^a^ 923 1484	131–131 428–626 824–956 1451–1715	−2.30 −0.85 −0.81 +0.88	6.37 {58%}	0.41
10	13.67	4	591 921 ^a^ 1350 1680	129–657 888–954 1284–1383 1680–1680	+0.86 −1.42 −1.18 +1.73	10.62 {78%}	0.48

**Figure A1:**
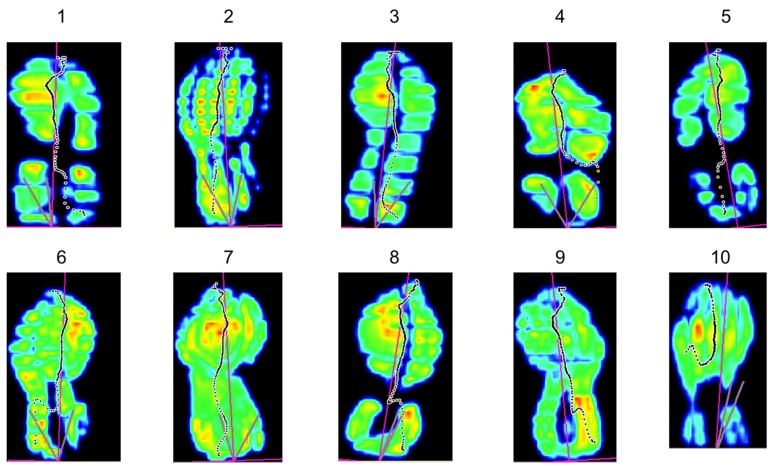
A peak pressure footprint for each subject (numbered 1 to 10) in the no-biofeedback condition. The centre of pressure path is indicated as a dotted line.

## Supplementary Materials

Supplemetary materials are available on the following link: https://www.mdpi.com/1424-8220/20/6/1720/s1. Video fragment of a subject wearing the biofeedback system while running indoors on an athletic track. It can be observed how the test leaders held supervision on a lap-by-lap basis.

## Appendix A

Determination of the foot strike pattern. Plantar pressures captured during the no-biofeedback condition to estimate the foot strike pattern (Figure A1). A 2-m pressure plate (Footscan; RSscan International, Olen, Belgium; sampling rate: 500 Hz; threshold value: 2.7 N·cm^-2^) with a newly replaced and factory-calibrated pressure-sensitive layer permitted us to estimate the foot strike pattern based on the instant of initial contact in the warm-up period. A rearfoot strike was defined when the first frame of the center of pressure occurred in the rear one-third of the foot–shoe system, while a non-rearfoot striker first touches the ground more anteriorly. The plantar pressures of three running trials were inspected. This resulted in nine runners who performed a rearfoot strike pattern and one performing a non-rearfoot strike. Representative plantar pressure images are shown.

## Appendix B

The change-point analysis is based on a statistical mean-shift model. It relies on the assumption of independence of errors around a possible changing mean. Since mean-shifted data are auto-correlated by definition, the software contains a pattern test to distinguish between shifts around a mean and autoregressive data [37]. A proposed workaround for correlated data is the grouping of data points. We were able to fulfil the assumptions for all subject data by grouping and averaging over 33 data points. This limited our temporal analysis to blocks of 33 axial peak tibial accelerations, which corresponded to approximately 25 s. Before detecting any change points, we needed to set a boundary for its confidence level. A change point can be detected by the use of a bootstrapping analysis on the cumulative sum (CUSUM) of the differences between the values of axial peak tibial acceleration and their average. A value D0 was calculated as the difference of the maximum and the minimum of the CUSUM. The peak tibial acceleration data were then reordered in a large number N of bootstrap samples. For these bootstrap samples, the difference of the maximum and minimum of the CUSUM was again calculated and the number of times X that this value was smaller than the original D0 was counted. The confidence level for detecting a change in the original sample was then defined as 100 × X/N%. This confidence level value has to be above the set boundary to detect a change point. The rationale is that in no-change conditions, where all data points are assumed interchangeable and which is simulated by the bootstrapping, a build-up of the CUSUM away from the zero value is less probable. The time of the change can be calculated by minimizing, over all possible change points, the mean square error of the data compared to a simple model made up of the two estimated averages of the data up to and after the possible change point. Confidence intervals for the timing of the change point were also calculated. After a change point was detected, the same analysis was done on the two data segments that were split by the change point. This was done until change points were no longer found, given the set confidence level boundary. After the complete set of change points was defined, all change points and their confidence levels were re-estimated [25]. Analogously, the change-point analysis was performed on the differences between consecutive grouped points to evaluate the variation on the time series [25].
